# Seoul orthohantavirus evades innate immune activation by reservoir endothelial cells

**DOI:** 10.1371/journal.ppat.1012728

**Published:** 2024-11-25

**Authors:** Stefan D. Klimaj, Autumn LaPointe, Kimberly Martinez, Eduardo Hernandez Acosta, Alison M. Kell

**Affiliations:** Department of Molecular Genetics and Microbiology, University of New Mexico School of Medicine, Albuquerque, New Mexico, United States of America; Johns Hopkins Bloomberg School of Public Health, UNITED STATES OF AMERICA

## Abstract

Pathogenic hantaviruses are maintained world-wide within wild, asymptomatic rodent reservoir hosts, with increasingly frequent human spillover infections resulting in severe hemorrhagic fever or cardio-pulmonary disease. With no approved therapeutics or vaccines, research has, until recently, focused on understanding the drivers of immune-mediated pathogenesis. An emerging body of work is now investigating the mechanisms that allow for asymptomatic, persistent infections of mammalian reservoir hosts with highly pathogenic RNA viruses. Despite limited experimental data, several hypotheses have arisen to explain limited or absent disease pathology in reservoir hosts. In this study, we directly tested two leading hypotheses: 1) that reservoir host cells induce a generally muted response to viral insults, and 2) that these viruses employ host-specific mechanisms of innate antiviral antagonism to limit immune activation in reservoir cells. We demonstrate that, in contrast to human endothelial cells which mount a robust antiviral and inflammatory response to pathogenic hantaviruses, primary Norway rat endothelial cells do not induce antiviral gene expression in response to infection with their endemic hantavirus, Seoul orthohantavirus (SEOV). Reservoir rat cells do, however, induce strong innate immune responses to exogenous stimulatory RNAs, type I interferon, and infection with Hantaan virus, a closely related hantavirus for which the rat is not a natural reservoir. We also find that SEOV-infected rat endothelial cells remain competent for immune activation induced by exogenous stimuli or subsequent viral infection. Importantly, these findings support an alternative model for asymptomatic persistence within hantavirus reservoir hosts: that efficient viral replication within reservoir host cells may prevent the exposure of critical motifs for cellular antiviral recognition and thus limits immune activation that would otherwise result in viral clearance and/or immune-mediated disease. Defining the mechanisms that allow for infection tolerance and persistence within reservoir hosts will reveal novel strategies for viral countermeasures against these highly pathogenic zoonotic threats.

## Author summary

Despite the significant, and continual, threat to human health, limited experimental evidence explains the mechanisms that underlie asymptomatic zoonotic RNA virus persistence within natural, mammalian reservoir hosts. Here, we investigated whether reservoir host target cells for hantavirus infection are competent for antiviral activation and tested the hypothesis that, through long-term co-evolution, Seoul orthohantavirus actively antagonizes innate antiviral signaling pathways to limit immune induction and prevent viral clearance in primary reservoir cells. While we find no evidence to support these hypotheses, our findings do support an alternative hypothesis that controlled viral replication within the natural reservoir cells may not result in the production of immune-stimulating by-products. The factors that determine viral persistence within the reservoirs warrant further investigation, but may include efficient use of host co-factors for efficient genome replication and/or packaging.

## Introduction

Zoonotic RNA viruses pose ever-present threats to human health. Maintained within natural reservoir host populations, they have the potential for human spillover and disease with rising frequency due to intensifying habitat encroachment and climate change. Viral dynamics within natural reservoir populations provide ecological clues for viral maintenance that can be applied to estimate the threat of zoonotic events [1–5]. Often zoonotic RNA viruses cause little-to-no disease within natural reservoirs, even in the presence of high viral loads [6–11]. Two leading hypotheses to explain host-specific infection outcomes include reservoir host disease tolerance due to heightened or dampened general immune responses and co-evolved virus-host interactions that antagonize immune signaling pathways [12–14]. In fact, different viral species likely employ unique mechanisms to modulate reservoir host responses toward the goal of persistence. Defining these mechanisms for mammalian reservoir hosts against viruses that otherwise cause severe disease in humans may shed light on the factors that drive pathogenesis, limit viral replication, and facilitate viral maintenance within reservoir populations.

Viral detection and the initiation of type I and type III interferon (IFN) responses within infected cells are critical to initiate an effective antiviral response capable of restricting viral replication, limiting further tissue dissemination, and facilitating viral clearance [15]. Double-stranded RNAs (dsRNA), highly structured RNAs, and unprocessed RNAs, which are recognized by the cytosolic RIG-I-like receptors (RLR), retinoic acid-induced gene I (RIG-I) melanoma differentiation-associated protein 5 (MDA5), drive transcription of antiviral effectors and types I and III IFNs [15–19]. The critical importance of this pathway in constraining viral replication and transmission is evidenced by almost universal antagonism of type I IFN signaling or effector function by successful viral pathogens [18]. Notably, while the intended outcome of type I IFN induction during infection is viral clearance, strong and sustained IFN responses are implicated in inflammatory disease, cancers, and significant tissue damage following viral infection [20–25]. Tight regulation of these pathways by the host allows for efficient antiviral response with limited tissue damage. Similar selective pressures also apply to viruses for which it would be beneficial to limit host immune activation to evade restriction and establish a replicative niche for long-term persistence and transmission.

The family Hantaviridae (Order *Bunyavirales*) is composed of insectivore- and rodent-borne viruses, maintained within reservoir populations through direct transmission [26–28]. Human infection with rodent-borne orthohantaviruses (here referred to as hantaviruses) occurs through inhalation of excreta from infected reservoirs, with endothelial cells (EC) being the primary cellular target for replication. Currently, no FDA or EU-approved vaccines or therapeutics exist that specifically target hantaviruses, despite case fatality rates between 1–60%, depending on viral species and outbreak. Severe human hantavirus disease is characterized by high levels of circulating proinflammatory cytokines (IL-6, TNFα), thrombocytopenia, and vascular leakage [29–32]. Hantaviruses are non-cytopathic and, in fact, inhibit both intrinsic and extrinsic apoptotic pathways in human EC [33–35]. Therefore, this robust inflammatory response to hantavirus infection is thought to be a central driver of tissue damage and severe human disease [31,36,37]. We previously reported that the RLRs are essential for interferon stimulated gene (ISG) expression in Hantaan orthohantavirus (*Orthohantavirus hantanense*, HTNV)-infected human umbilical vein endothelial cells (HUVEC) [38]. In wild-type HUVEC, we observed robust ISG expression beginning 48hrs post-infection, which was delayed in RIG-I^-/-^ cells and ablated in the absence of both RLR. We further found that UV-inactivated HTNV did not drive ISG induction, supporting the hypothesis that viral replication intermediates are the primary pathogen-associated molecular patterns (PAMPs) for hantaviruses in human cells. Importantly, humans are a dead-end host for hantaviruses, with extremely rare exceptions for Andes orthohantavirus, and the antiviral response can be effective for viral clearance in survivors and fatal victims of hantavirus infection [39–42]. Whether sustained activation of type I IFN responses in human EC serves as the catalyst of an unchecked systemic inflammatory syndrome and severe hantavirus disease, despite apparent viral control, remains to be explored.

In contrast, *in vivo* hantavirus infection in natural reservoir hosts leads to a very mild systemic antiviral response early, followed by a dominant regulatory T cell signature, despite high viral loads [43–46]. Importantly, individual hantavirus species are closely associated with specific mammalian reservoir hosts, leading to a hypothesis of extended co-evolution between virus and reservoir host [28,47]. For example, Sin Nombre orthohantavirus is found almost exclusively within *Peromyscus* (Deer mouse) species in the Americas, HTNV has been isolated from *Apodemus agrarius* (striped field mouse) in Asia, and Seoul orthohantavirus (*Orthohantavirus seoulense*, SEOV) has been identified in *Rattus* species (predominantly Norway rat) throughout the world [48–53]. Previous work by several groups has established a paradigm in which infection of natural hantavirus reservoir rodents with their endemic, potentially co-evolved, virus species results in asymptomatic persistent infection, whereas infection of that rodent species with a non-endemic hantavirus leads to rapid antiviral activation and viral clearance [54–57]. *In vitro* infections of reservoir endothelial cells with their endemic hantavirus do not stimulate antiviral gene expression and mount a mild, if any, innate immune response to their endemic hantavirus [58, 59]. However, the mechanisms that underlie immune recognition and infection outcome for each unique virus-host relationship are poorly defined.

Although tools to identify novel zoonotic viruses and to systematically document reservoir prevalence have rapidly improved in recent decades, the virus-host interactions that allow for asymptomatic virus persistence within these natural hosts remain elusive. Several hypotheses to explain persistence within reservoir hosts have been put forth, with a few becoming widely accepted, despite limited experimental support [12–14]. The co-evolved nature of human pathogenic hantaviruses with their reservoir rodent hosts provides us with a unique opportunity to test these hypotheses at the cellular level. Here, we describe robust innate immune activation and inflammatory responses in human EC in response to SEOV infection that are dependent on RIG-I-like receptor activity. In contrast, we show that primary reservoir rat EC do not mount an antiviral ISG response to their endemic hantavirus, SEOV, despite robust infection and replication. We show that rat lung microvascular endothelial cells (RLMVEC) upregulate ISGs in response to non-endemic hantavirus infection, type I IFN, and known RLR agonists. We rigorously tested the hypothesis that SEOV directly antagonizes innate immune signaling pathways by interfering with RLR or type I IFN signaling and found no evidence for it. The studies presented here instead suggest a more complex hypothesis: that endemic hantaviruses may replicate more efficiently within reservoir target cells to prevent immune recognition and antiviral activation. Defining the mechanisms employed by zoonotic viruses and their hosts to regulate immune-mediated damage induced by infection will reveal novel strategies for therapeutic development against severe human disease.

## Results

### Reservoir and human EC are differentially susceptible to SEOV infection

**Fig 1:**
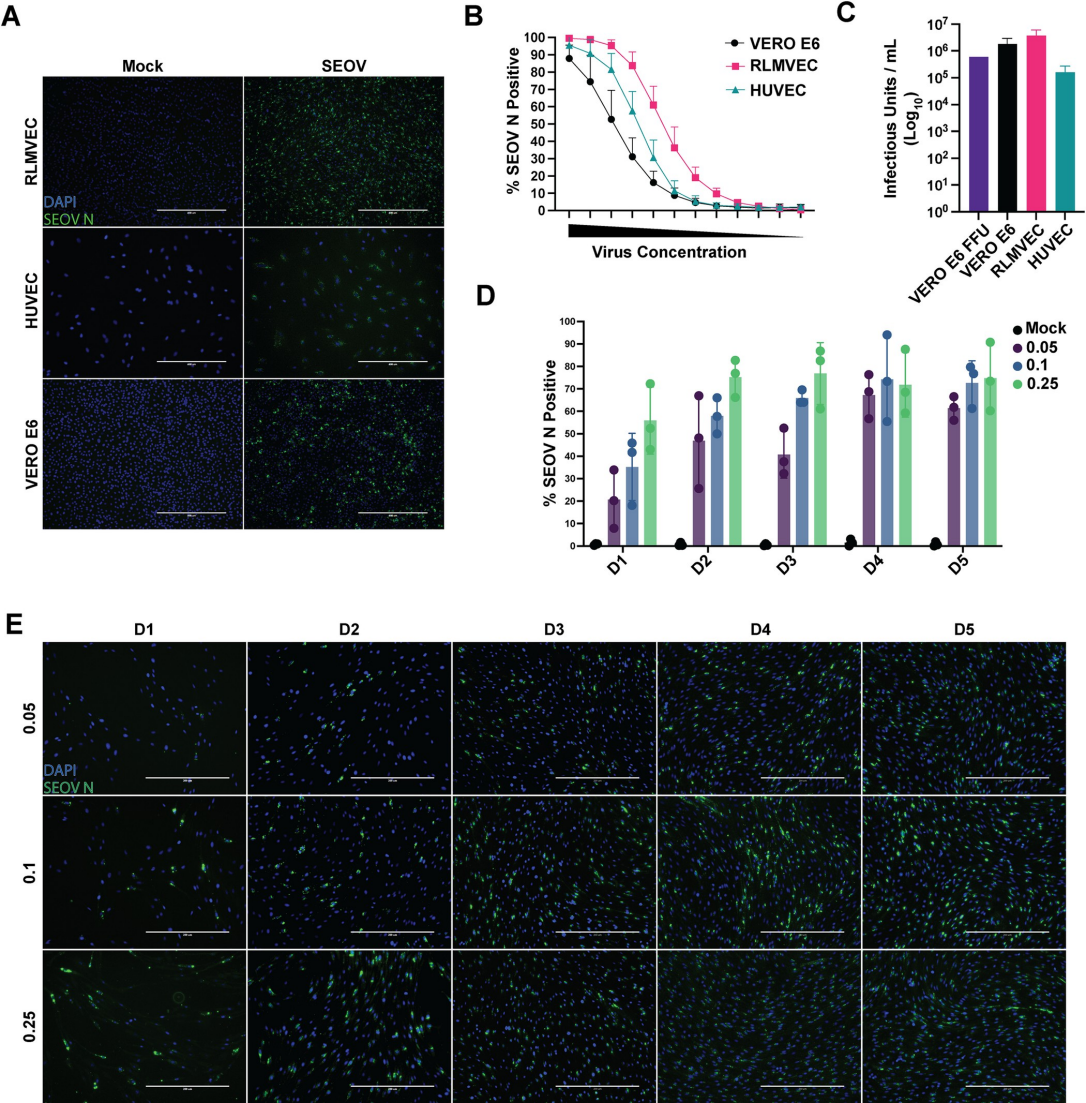
Quantification of SEOV infection in human and rat endothelial cells. A) Representative images of mock or SEOV-infected RLMVEC, HUVEC, and Vero E6 cells (1,8 dilution) at 10x magnification. Nuclei are stained with DAPI (blue) and SEOV N protein is shown in green. B) Percentage of SEOV N-positive cells at each 1:2 viral dilution from neat to 1:1024 dilution and mock to determine background fluorescence, quantified using the CellInsight CX7 imaging platform for all cell types. C) Calculated infectious units/mL for each cell type based on focus-forming unit (FFU) assay for Vero E6 and CellInsight CX7 imaging for Vero E6, RLMVEC and HUVEC (mean ± SD, from four replicate wells per dilution, across three independent experiments). D) Percentage of SEOV N-positive RLMVEC over five days, following infection with MOIs of 0.05, 0.1, and 0.25, quantified using CellInsight CX7. E) Representative images of SEOV-infected RLMVEC at each MOI and timepoint, captured at 20x magnification.

It has been previously shown that both HUVEC and RLMVEC are susceptible to SEOV infection, however a quantitative assessment of SEOV infection in comparison to immune-incompetent Vero E6 cells has not been reported. Because hantaviruses are non-cytopathic, a focus-forming unit assay, reliant on antibody staining for the viral nucleocapsid (N) protein, in Vero E6 cells is the established method for titering virus stocks [60]. However, primary cells are less tolerant of the seven day incubation period and methylcellulose overlay required for this assay, making it challenging to determine viral titer on relevant, immune-competent cells. We therefore developed a novel immunofluorescence assay for titering our viral stocks on Vero E6, RLMVEC, and HUVEC based on the detection of SEOV N protein (**Fig 1A**). Using the Thermo Scientific CellInsight CX7 High-content imaging platform we quantified the percentage of N+ cells at each dilution of virus stock from undiluted to 1:1024 (**Fig 1B**) and applied the methodology proposed by Menke and colleagues to determine the infectious unit count/milliliter (mL) [61]. Applying this equation to our quantification results derived from microscopy, we were able to quantify the cell-specific infectious units/mL in our virus stock (**Fig 1C**). We calculated a similar number of infectious units/mL on Vero E6 cells using both the traditional FFU assay (6x10^5^) and our novel microscopy assay (2x10^6^). Importantly, we determined that the number of infectious units within the same virus stock in RLMVEC was only slightly higher than that for Vero E6 cells, suggesting that both cell types are equally susceptible to virus infection. Conversely, the number of infectious units/mL quantified for HUVEC was more than a log lower than Vero E6 or RLMVEC, indicating that these cells are less susceptible to SEOV infection. Notably, although a greater percentage of HUVEC were infected with SEOV at several dilutions, compared to Vero E6, HUVEC are much larger and fewer individual cells were plated in each confluent well, therefore the total number of HUVEC infected was lower than Vero E6. We report here the multiplicity of infection (MOI) in subsequent experiments based on these cell-type specific titers.

To quantify the percentage of infected cells over a spreading infection, we infected RLMVECs with SEOV at MOI of 0.05, 0.1, and 0.25, assessing the proportion of SEOV N-positive cells at each timepoint. As expected, the low MOI of 0.05 led to a lower initial infection rate, but the infection steadily propagated, reaching approximately 60% of cells by day five. In contrast, the higher MOIs (0.1 and 0.25) resulted in infection rates of 70–90% by the end of the experiment (**Fig 1D and 1E**). Based on these results, we selected an MOI of 0.05 for subsequent experiments, as it reflects a likely physiologically relevant viral load during natural infection while still achieving a sufficiently high infection rate to assess potential innate immune antagonism.

### Seoul virus infection drives RIG-I-dependent ISG expression in HUVEC

**Fig 2:**
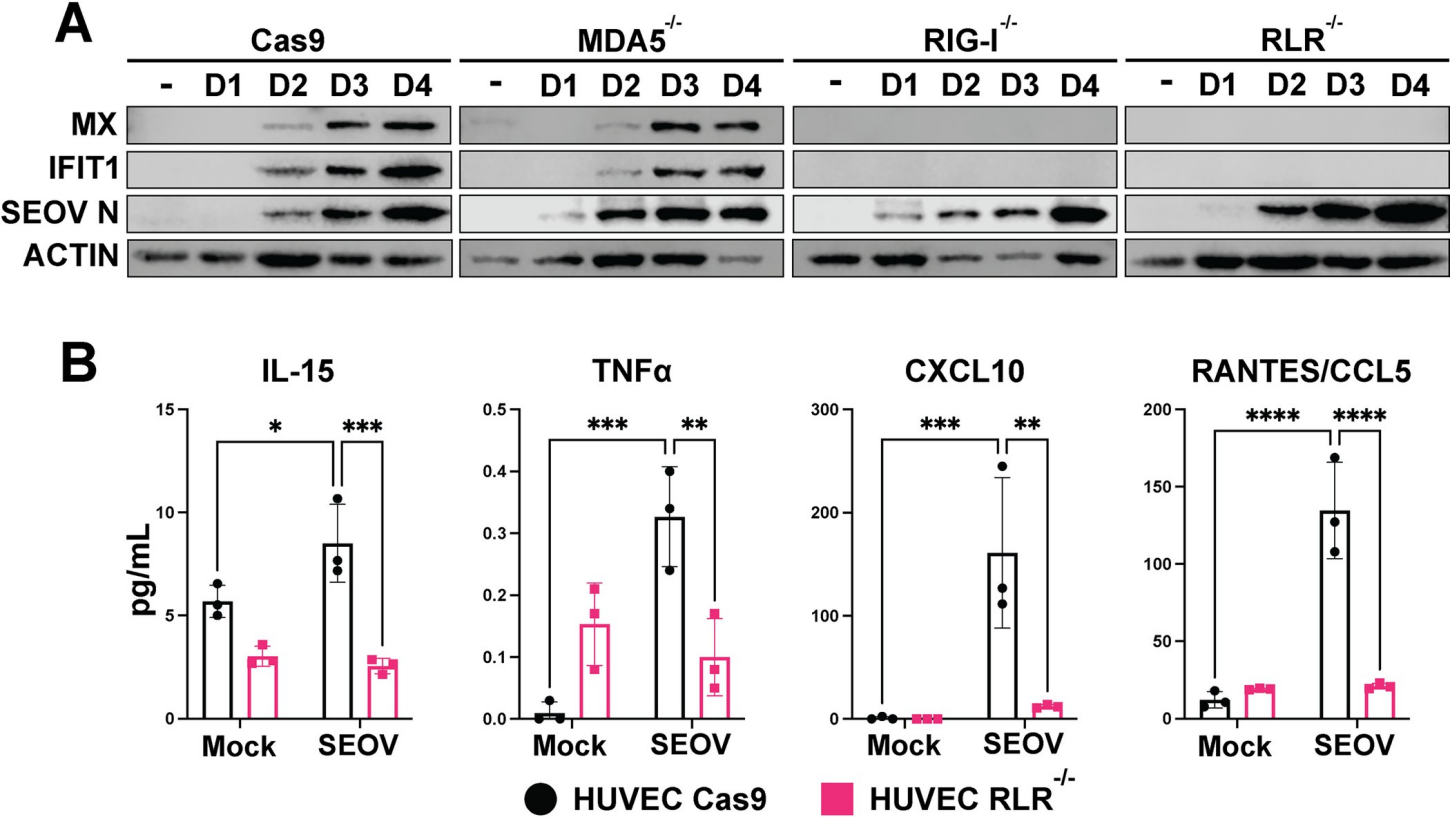
SEOV-induced ISG expression in HUVEC is RIG-I-dependent. CRISPR-modified HUVEC (Cas9 alone, RIG-I^-/-^, MDA5^-/-^, or both RIG-I-like receptors (RLR^-/-^) were infected with SEOV at MOI 0.01. Cell lysates and supernatant were collected every 24 hours for four days. A) Cell lysates were run on SDS-PAGE and subjected to immunoblot analysis. Data shown are representative of three independent experiments. Densitometry quantification of immunoblots in S2 Fig. B) Supernatants of SEOV-infected cells were collected on day four and analyzed for cytokine and chemokine levels. Data represent three biological replicates averaged across technical replicates ± SD. Two-way ANOVA with multiple comparisons, * denotes adjusted p<0.05.

To determine whether the RLRs are required for recognition of, and immune signaling against, SEOV, we infected HUVEC lacking either RIG-I or MDA5, or both RLRs at MOI 0.01. Similar to our HTNV observations, SEOV infection in Cas9 control cells led to increased ISG expression over time, concomitant with SEOV nucleocapsid detection (**Fig 2A**) [38]. We observed that RIG-I^-/-^ and RLR^-/-^ HUVEC lacked all ISG induction following SEOV infection, with MDA5^-/-^ resembling the wild-type control. We next asked whether SEOV infection induces inflammatory cytokine and chemokine signaling in an RLR-dependent fashion. WT HUVEC or RLR^-/-^ cells were infected with SEOV and supernatant was interrogated for secreted cytokines and chemokines four days post-infection (**Figs 2B and S1**). As expected, we observed an increase in neutrophil, monocyte, and NK cell recruitment cytokines CXCL10, CCL5, and IL-15 during SEOV infection in Cas9 WT control HUVEC. This induction was lost in SEOV-infected RLR^-/-^ HUVEC. These results suggest that RLR signaling in response to SEOV infection in human EC may also catalyze a larger proinflammatory state within the vasculature through chemokine secretion and endothelial activation.

### Seoul virus infection in primary rat EC does not drive ISG expression

**Fig 3:**
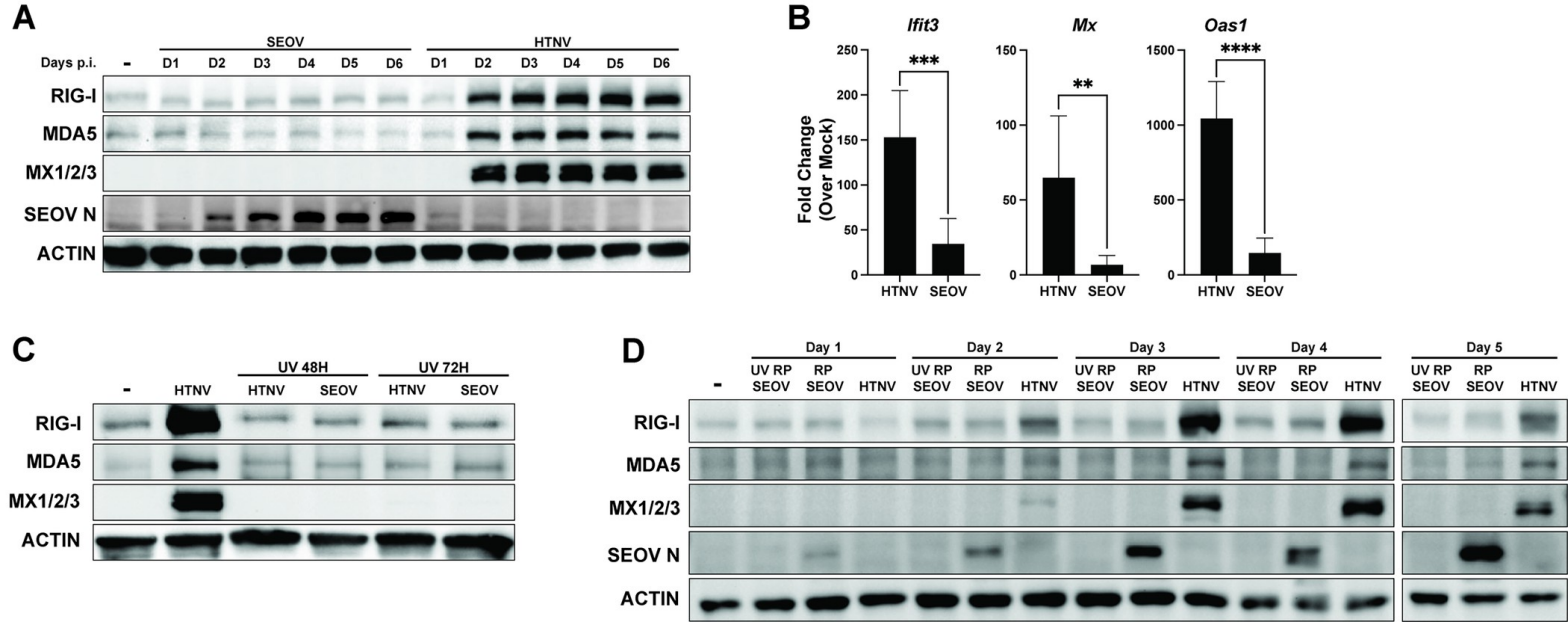
Primary rat EC induce ISGs in response to non-endemic hantavirus, HTNV, but not to endemic hantavirus, SEOV. A) RLMVEC were infected with either SEOV or HTNV (MOI 0.05) or mock infected (-) and lysates were collected every 24 hours for six days (D1, D2, etc.) post-infection. Cell lysates were subjected to SDS-PAGE and immunoblot analysis for ISG expression. B) RLMVEC were infected with either SEOV or HTNV (MOI 0.05) and harvested 48 hours post-infection. Gene expression was assayed by comparative RT-PCR and averaged data from technical replicates and three experimental biological replicates are shown. Student’s T test, * denotes p<0.05. C) RLMVEC were infected with either HTNV or UV-inactivated HTNV or SEOV (MOI 0.05). Lysates were harvested at the indicated times post-infection and subjected to immunoblot analysis. D) RLMVEC were infected with either RLMVEC-passaged SEOV (RP SEOV) (MOI 0.025), UV-inactivated RLMVEC-passaged SEOV (UV RP SEOV) (0.025), or HTNV (MOI 0.025) and lysates were harvested at the indicated times post-infection and subjected to immunoblot analysis. Densitometry quantification of immunoblots in S4 Fig. All data shown are representative of ≥3 independent experiments.

Next, we interrogated reservoir target cells for antiviral responses to endemic and non-endemic hantavirus infection. We infected RLMVEC with either SEOV (endemic hantavirus) or HTNV (closely related, but for which rats are not the reservoir host) at 0.05 MOI **(Fig 3A)**. While HTNV-infected cells increased expression of the ISGs RIG-I, MDA5, and Mx1/2/3 over the course of the six-day infection, SEOV did not drive increased ISG protein production. Similar results were observed at the transcript level on day two post-infection for antiviral genes *Ifit3*, *Mx1*, and *Oas1*, with SEOV inducing minimal gene expression **(Fig 3B)**. Importantly, as we have observed previously in HUVEC, UV-inactivated HTNV did not induce ISG expression in these cells, indicating that antiviral signaling requires viral replication **(Fig 3C)** [38]. To confirm that these results are not strain-specific, we infected primary RLMVEC with the Baltimore strain of SEOV with consistent observations (**S3A Fig**) [62]. Previous reports have indicated a potential loss of pathogenicity for hantaviruses following culture in Vero E6 cells [63,64]. Therefore, we passaged our SR-11 strain of SEOV exclusively in RLMVEC three times and then assessed ISG expression in RLMVEC (**Fig 3D**). Importantly, we observed no ISG induction following infection of RLMVECs with any SEOV strains, while strong ISG induction was noted with HTNV infection. As a control, UV-inactivated SEOV was used to assess whether cytokines produced during SEOV propagation on primary rat cells could drive ISG expression, but no such induction was observed. These findings demonstrate that primary rat cells mount antiviral responses to the non-endemic hantavirus HTNV but fail to respond to SEOV infection, even with sustained viral replication.

### Primary rat EC maintain intact antiviral responses to exogenous RNA stimuli

**Fig 4:**
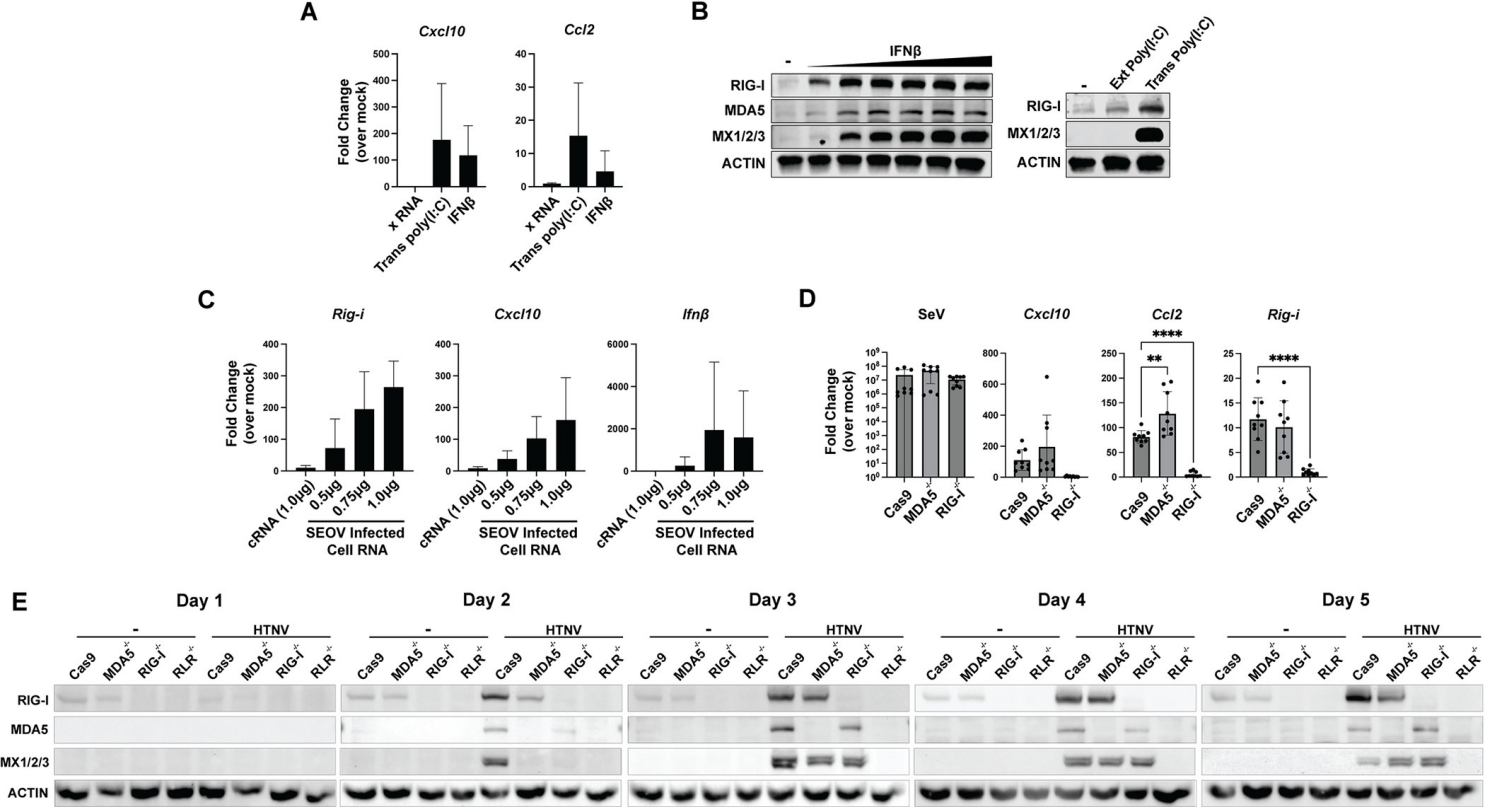
Primary RLMVEC induce antiviral responses to canonical signaling agonists. A, B) RLMVEC were either treated with exogenous recombinant rat IFNβ (1-100U/mL) or transfected with HCV-derived X RNA or poly(I:C) (100ng). RNA (18 hours) and lysates (24 hours) were collected post-treatment and subjected to comparative RT-PCR analysis (A) or immunoblotting (B). C) RLMVEC were transfected with indicated amounts of total RNA isolated from either mock-infected Vero E6 cells (cRNA) or SEOV-infected Vero E6 cells (icRNA). RLMVEC RNA was collected 18 hours post-transfection for comparative RT-PCR analysis, data presented represent the average of three biological replicates, with technical replicates averaged within each experiment. Densitometry quantification of immunoblots in S6 Fig. All data shown represent ≥3 independent experiments, ±SD.

Altered immune function or tolerance are popular explanations for the seemingly unique ability of rodent and bat reservoir hosts to support significant and diverse pathogen burden [6–8,10]. Therefore, we investigated the ability of primary RLMVEC to induce antiviral gene expression following treatment with known stimulatory RNA and exogenous type I interferon. Rat ECs transfected with poly(I:C), a dsRNA mimic which activates RLR-dependent signaling, or treated with recombinant rat IFNβ, increased relative transcription of *Cxcl10* and *Ccl2* compared to mock-treated controls **(Fig 4A).** X RNA is an *in vitro* transcribed 100-nucleotide sequence derived from the hepatitis C virus genome that does not drive RLR activation and serves as an additional control [65,66]. Increased expression at the protein level for RIG-I, MDA5, and Mx1/2/3 was also observed for RLMVEC treated with exogenous IFNβ and transfected poly(I:C) (**Fig 4B)**. Curiously, we did not detect ISG expression following addition of poly(I:C) to the cell culture media, which has been reported to stimulate TLR3 signaling [67]. We next asked whether RNA PAMPs created during SEOV infection could stimulate an antiviral response in the absence of viral proteins. To test this, we isolated total RNA from vero-infected RLMVEC five days post-infection, capturing all host and viral RNAs present. Total RNA from uninfected RLMVEC (cRNA) was also isolated as a negative control. We then transfected the infected-cell RNA (icRNA) into uninfected RLMVEC and measured relative antiviral gene expression 18 hours later. We observed a dose-dependent increase in ISG transcription following transfection of icRNA when compared to cells transfected with cRNA, although variation in transfection efficiencies prevented statistical significance **(Fig 4C)**. We generated CRISPR knockout lines of primary RLMVECs for RIG-I, MDA5, or both RLRs to assess receptor dependence in PAMP sensing and subsequent innate immune induction (**S5 Fig**). Sendai virus (SeV), a paramyxovirus, selectively activates RIG-I through the production of 5’ triphosphate RNA during replication [68]. We validated that RLMVECs respond canonically to SeV by infecting RLMVECs lacking either RIG-I or MDA5 with 150 HAU/mL for 24 hours, confirming RIG-I-dependent signaling (**Fig 4D**). We have previously demonstrated that HTNV is recognized through both RIG-I and MDA5 in human and mouse cells [38]. To determine whether this was also true for RLMVEC, we infected our knockout lines with HTNV at an MOI of 0.05 for five days (**Fig 4E**). We observed ISG expression in non-target control cells comparable to the single RLR knockout lines. However, cells lacking both RIG-I and MDA5 failed to induce ISG expression, demonstrating that HTNV is recognized by both receptors. Together, these data demonstrate that primary RLMVEC are immune-competent and respond canonically to known RLR agonists. Further, SEOV RNA replication intermediates, or other viral-induced host RNAs, stimulate an innate immune response in reservoir EC in the absence of viral proteins.

### Active SEOV infection does not antagonize antiviral gene expression in primary rat EC

**Fig 5:**
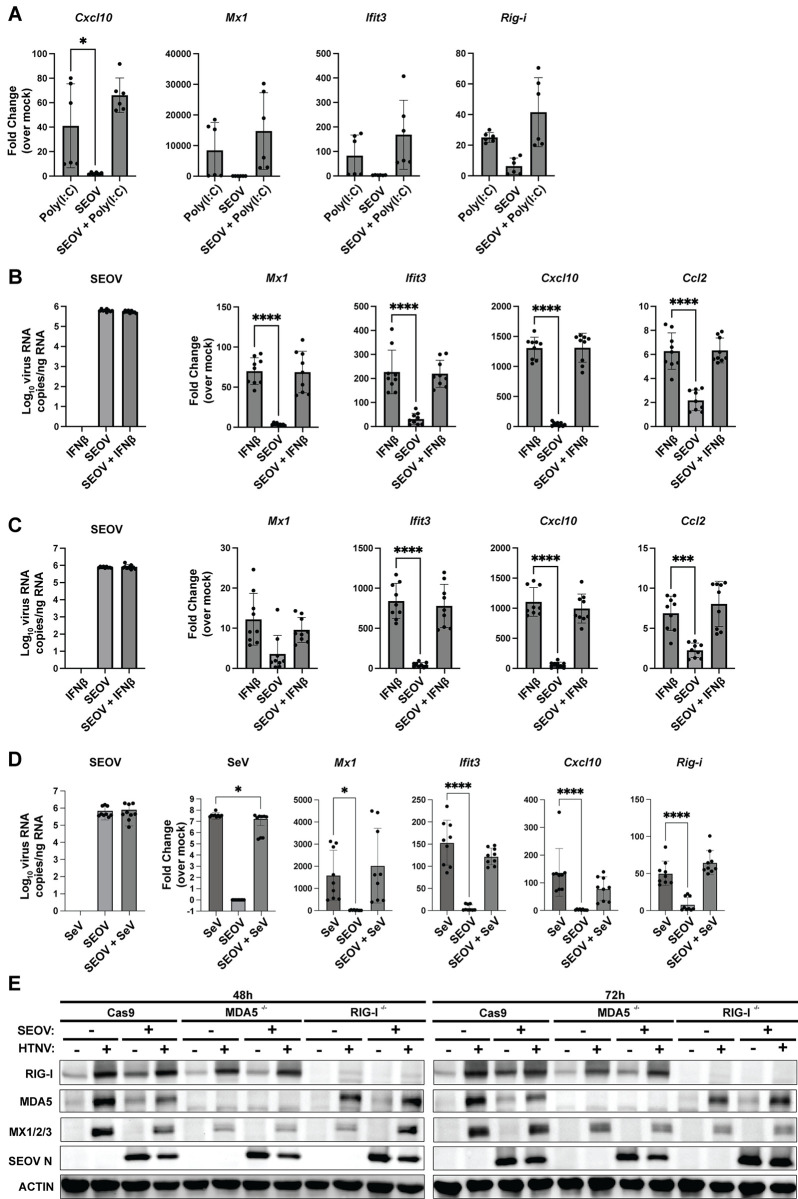
SEOV infection does not inhibit subsequent ISG induction. A) RLMVECs were mock-infected or infected with SEOV (MOI 0.1) for 72 hours, followed by transfection with poly(I:C) (100 ng). RNA was collected 24-hours post-treatment to assess ISG expression. B-C) RLMVECs were mock-infected or infected with SEOV (MOI 0.1) for 48 hours (B) or 72 hours (C), followed by IFNβ treatment (100 U/mL). RNA was collected at 24 hours post-treatment to evaluate ISG induction. D) RLMVECs were mock-infected or infected with SEOV (MOI 0.05) for 5 days, followed by either mock infection or superinfection with SeV (150 HAU/mL). RNA was collected 24 hours after SeV infection to measure viral genome copies and ISG expression using qRT-PCR and comparative RT-PCR, respectively. E) RLMVEC Cas9 scramble, RIG-I^-/-^, or MDA5^-/-^ cells were either mock-infected or infected with SEOV (MOI 0.05) 48 hours. Cells were the either subsequently superinfected with HTNV (MOI 0.05) or mock-infected. Lysates were collected at the indicated times post-HTNV infection and subjected to SDS-PAGE and immunoblot analysis. Densitometry quantification of immunoblots in S7 Fig. All data shown represent ≥3 independent experiments, with technical replicates averaged within each experiment ±SD. One-way ANOVA with multiple comparisons to mock-infected poly(I:C), IFNβ, or SeV-treated control (* denotes p<0.05).

**Fig 6:**
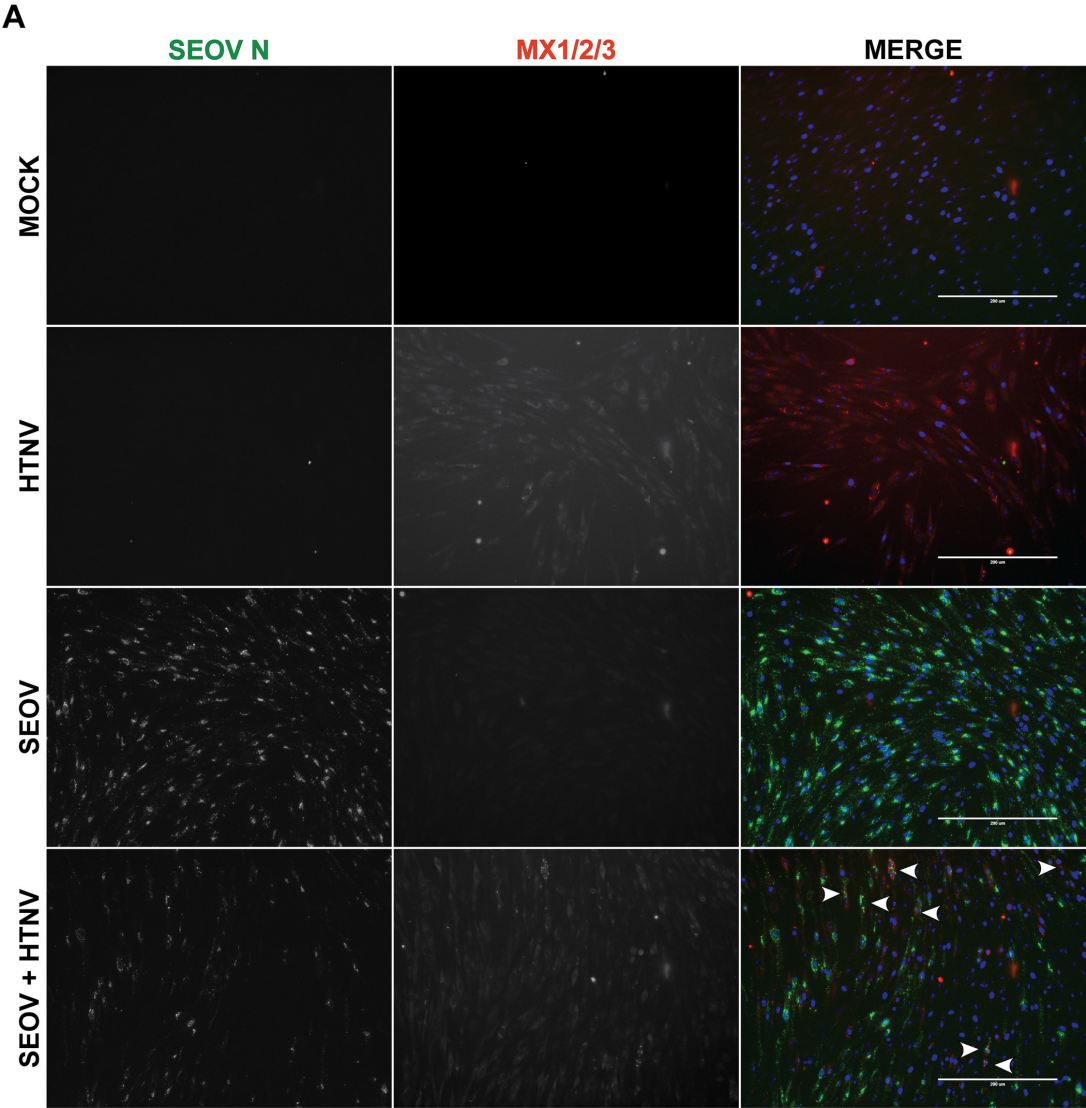
SEOV does not inhibit HTNV-induced Mx1/2/3 expression. RLMVEC were either mock-infected or infected with SEOV (MOI 0.05) for 48 hours. Cells were subsequently either mock-infected or superinfected with HTNV (0.05 MOI) and fixed 48 hours post-HTNV infection. Images of immunostaining for SEOV nucleocapsid (green), MX1/2/3 (red), and DAPI (blue). Images were captured on an EVOS FL Auto imaging system using 20x magnification. Arrows indicate individual cells expressing both Mx1/2/3 and SEOV N. Images are representative of 2 independent experiments.

Because immune-competent RLMVEC respond to viral and/or viral-induced RNA in the absence of viral proteins but do not induce ISGs during active SEOV infection, we hypothesized that SEOV proteins may inhibit RLR signaling or autocrine type I IFN activation in reservoir rat cells. To test this hypothesis, we infected RLMVEC with SEOV at MOI 0.05, allowed the virus to replicate and produce viral proteins for 72 hours, and then transfected poly(I:C) (**Fig 5A**). If SEOV infection actively antagonizes RLR signaling, we would expect a reduction in ISG expression compared to uninfected, poly(I:C)-treated cells. Surprisingly we observed no reduction in ISG expression in the presence of prior SEOV infection. Next, we infected RLMVECs with SEOV for either 48 (**Fig 5B**) or 72 hours (**Fig 5C**), followed by IFNβ treatment to assess potential antagonism of IFNAR1 signaling. We observed no reduction in ISG induction by IFNβ in SEOV-infected cells compared to uninfected controls. To investigate the specific inhibition of RIG-I signaling by SEOV, RLMVECs were infected with SEOV (MOI 0.05) or mock-infected for 5 days, followed by either mock infection or superinfection with SeV (150 HAU/mL). SeV alone activated ISG transcription, and prior SEOV infection did not reduce this response, (**Fig 5D**). To test whether prior SEOV infection could block ISG expression through a single RLR, we infected our RLMVEC RLR KO lines, first with SEOV and then with HTNV. For example, if SEOV specifically counteracts the activation of rat MDA5, we would expect that HTNV would fail to induce ISG expression in SEOV-infectedRIG-I^-/-^ RLMVEC, due to a loss in functionality of both RLRs. In **Fig 5E**, we observe the lack of either RIG-I or MDA5 reduces the expression of Mx1/2/3 following HTNV infection. However, we observe no difference in ISG expression between cells infected with HTNV alone compared to cells first infected with SEOV and then HTNV in either non-target controls (Cas9) or KO cells. Finally, to confirm that HTNV-driven ISG expression was in fact occurring in SEOV-infected cells, we performed immunofluorescence assays probing for Mx1/2/3 protein expression 48hrs post-HTNV superinfection (**Fig 6**). By co-staining for SEOV nucleocapsid and Mx1/2/3, we observed that Mx signal was produced in SEOV N (+) cells (white arrows). Regrettably, a lack of specific antibodies against HTNV nucleocapsid prevents identification of HTNV-infected cells in this assay. However, this result demonstrates that SEOV infection does not limit the induction and signaling of type I IFN. We therefore conclude that direct antagonism of either RLR- or type I IFN-signaling pathways is not the mechanism by which SEOV is able to replicate in primary reservoir EC without triggering immune activation.

### Increasing SEOV MOI leads to RLR-dependent innate immune signaling in RLMVEC

**Fig 7:**
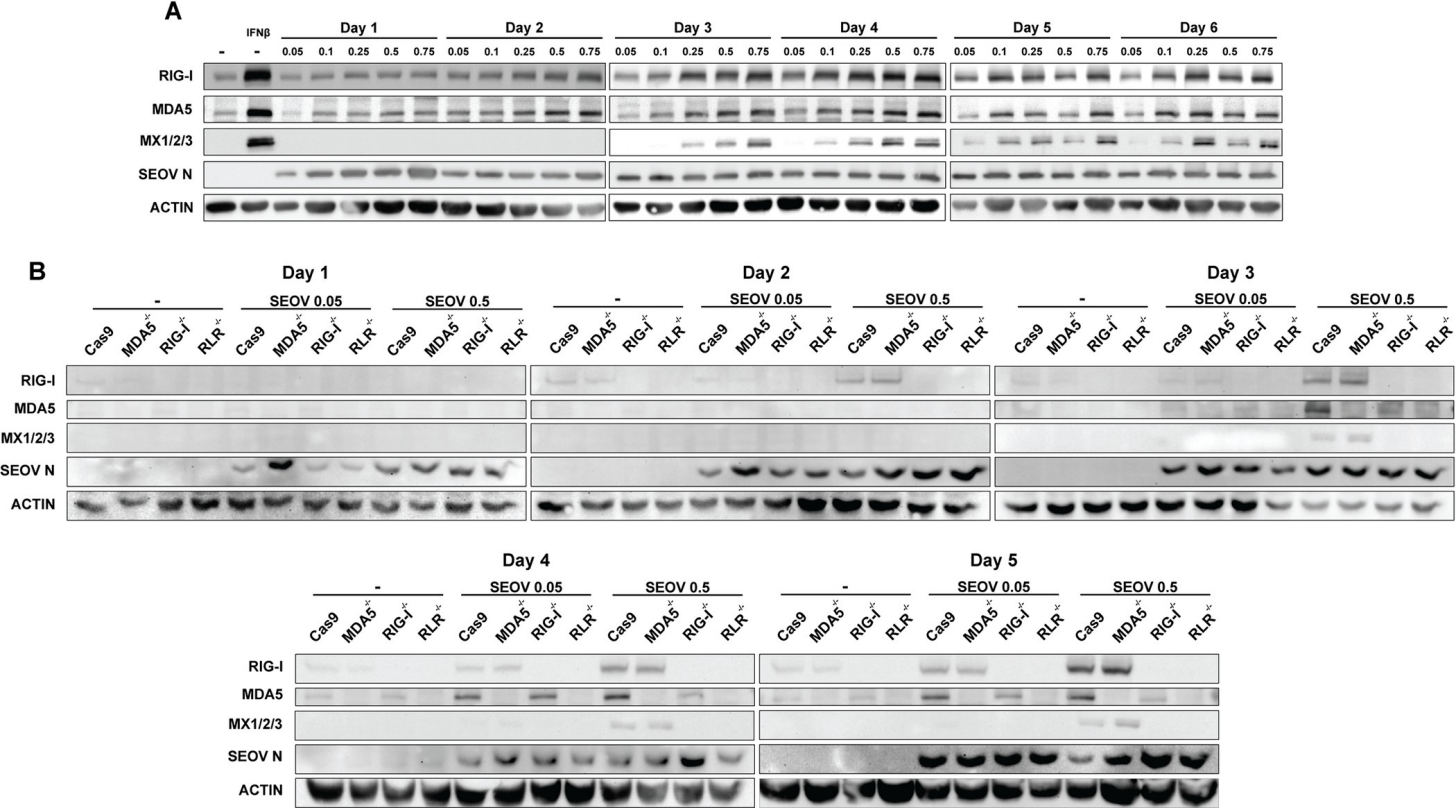
High MOI infections drive RLR-dependent ISG expression in RLMVEC. A) RLMVEC were infected with SEOV at the noted MOI 0.05–0.75 and cell lysates collected every 24 hours, as indicated. Cell lysates were subjected to immunoblot analysis. Mock-infected RLMVEC (-) treated with 100U/mL IFNβ 24 hours prior to harvest serves as a positive control. B) Cas9 scramble, RIG-I^-/-^, MDA5^-/-^, or RLR^-/-^ RLMVEC were mock-infected (-) or infected with SEOV at either MOI 0.05 or 0.5 and harvested at the indicated times post-infection. Lysates were subjected to immunoblot analysis. Densitometry quantification of immunoblots in Fig S8. All data shown represent ≥3 independent experiments.

Given these results, and the susceptibility of RLMVEC to SEOV infection, we tested whether increasing viral dose might drive antiviral activation in these reservoir cells. Western blot analysis revealed a dose-dependent increase in ISG expression with increasing MOI (**Fig 7A**). Notably, viral nucleoprotein is detected in all infection conditions, revealing that the number of incoming infectious units at the initial infection of the cell culture is what determines immune activation in these reservoir cells. We, therefore, hypothesized that high MOI infections may lead to increased production of defective viral genomes or accumulation of defective replication intermediates that may be recognized by RLRs to induce antiviral responses [69–72]. To test this hypothesis, we infected our CRISPR knockout primary RLMVEC, lacking RIG-I, MDA5 or both RLRs at a range of MOI. Across several experiments, we determined that RIG-I was exclusively required for ISG expression (**Fig 7B**). These findings again demonstrate that primary RLMVEC are competent for sensing viral infection, but that low MOI SEOV infections allow the virus to subvert antiviral activation despite productive spread in culture.

## Discussion

Our study investigated the potential cellular mechanisms underlying hantavirus persistence within their asymptomatic, natural reservoir hosts. We have presented evidence that primary rat reservoir target cells do not induce antiviral responses to low MOI infection with their endemic hantavirus, SEOV. This was observed despite high susceptibility to spreading infection and increasing viral protein expression, with low initial MOI. Our investigations further demonstrate that defects in RLR- or type I IFN-dependent signaling in RLMVEC do not explain this immune silence. Finally, we find no evidence of SEOV antagonism of these innate immune signaling pathways. Further work will be necessary to investigate alternative mechanisms of immune evasion such as RNA sequestration, replication efficiency, and metabolic regulation that may play a larger role in the establishment of persistence.

Immune activation, specifically an overactive proinflammatory response, is associated with severe hantavirus disease in humans [73]. We have now shown that both HTNV and SEOV drive robust antiviral signaling in human EC, and that this signaling is dependent on the RIG-I-like receptors (**Fig 2** [38]). Results from our RLR^-/-^ HUVEC line indicates that, while HTNV can be detected by both MDA5 and RIG-I, likely in a temporal-specific manner, antiviral responses to SEOV are uniquely RIG-I-dependent. Therefore, RLR-dependent innate immune activation may be a conserved human EC response to Old World hantavirus infections. Importantly, induction of ISG expression occurs in HUVEC despite relatively low levels of initial viral infection when compared to RLMVEC (MOI 0.01 vs 0.05 or 0.1, respectively, **Figs 1 and 2A**). HUVEC are likely, however, exposed to more viral particles per cell (0.1 vero-derived MOI). While we cannot rule out that this may play a role in immune activation, previous work has demonstrated that viral replication is required for immune activation in hantavirus infections [38]. These observations suggest that viral PAMPs may be more readily exposed during HUVEC infection, possibly due to poor use of host co-factors required for replication complex formation, mRNA cap-snatching, or incomplete virion assembly [72]. Additionally, robust RLR-dependent innate immune activation may set the stage for increased infiltration of immune cells, such as proinflammatory neutrophils or monocytes, which have been implicated in severe disease [29, 74].

We were surprised to observe a complete absence of ISG induction in primary RLMVEC following SEOV infection at MOI 0.05 even six days post-infection (**Fig 3A**). These experiments were performed with a low starting MOI (0.05) to represent likely physiologic conditions with transmission involving a small number of infectious particles [75]. Despite clearly increasing nucleocapsid accumulation on days three and four post-infection, our western blot analysis shows no ISG expression even out to six days post-infection. We would expect that, even if low early levels of infection did not produce a strong IFN signal *in vitro*, by six days post-infection any interferon secretion would drive the amplification of an antiviral response in infected and uninfected cells in culture. Thus, the lack of such a signature is noteworthy. Further, infection with a different strain of SEOV and RLMVEC-passaged SEOV resulted again in an absence of ISG expression, suggesting that this is not a unique artifact of strain history (**Figs 3D and S3A**). Infection of rat ECs with HTNV did induce robust ISG expression. Thus, reservoir rat EC mount an antiviral response to non-endemic HTNV infection, but not endemic SEOV. We also interrogated the transcriptional upregulation of *Rig-i*, *Cxcl10*, *Ccl2*, *Mx1*, and *Oas1* by RT-PCR (**Figs 3B and 5A–5D**). A scarcity of clean, reliable antibodies for western blotting has limited a deeper investigation for protein expression. However, it is notable that, by assessing both transcription and translation of antiviral genes and ISGs, we have shown that host translational shutoff is not likely to be a mechanism of immune evasion employed by SEOV in its reservoir. The ability of RLMVECs to translate ISGs when infected at high SEOV MOI also supports this conclusion.

Importantly, others have previously investigated antiviral responses by hantavirus reservoir cells to their endemic virus [58, 59, 76]. *In vitro* infections of ECs from Norway rats and bank voles have demonstrated that innate immune activation is muted in these cells [58,59]. It, therefore, may be a universal phenotype of hantavirus reservoirs to curb innate immune responses to their endemic viruses, representing a potential mechanism of persistence. Dampened immune signaling has been described for several species of bat, reservoirs of highly pathogenic viruses, such as other hemorrhagic fever viruses and rabies virus [8,10,77]. It is an especially appealing hypothesis to explain the ability of rodents and bats to harbor diverse viral species which otherwise cause immune-mediated pathologies in humans, driven by uncontrolled immune activation. We therefore interrogated the induction of ISGs in response to known agonists of the RLR pathway or type I IFN signaling. Both transfected poly(I:C) and recombinant rat IFNβ drove the transcription of ISGs *Ccl2*, *Cxcl10*, and *Rig-i* (**Fig 4A and 4B**). We further demonstrated that Sendai virus, and known RIG-I agonist, and HTNV both require intact RLR signaling for ISG induction (**Fig 4D and 4E**). We therefore have no indication that the Norwegian rat exhibits unique immune signaling responses and do not believe this explains the observed lack of ISG expression during SEOV infection.

Given that hantaviruses are thought to have co-evolved along with their rodent reservoir host, we hypothesized that SEOV has evolved mechanisms to directly antagonize antiviral pathways within its natural reservoir to maintain active replication without immune induction [28,47,78]. We tested whether, in the absence of viral proteins, viral RNAs present during infection are capable of driving innate immune activation. Transfection of total RNA isolated from SEOV-infected Vero E6 cells induced antiviral gene transcription in a dose-dependent manner, suggesting that viral proteins are somehow involved in either antagonizing the RLR pathway or shielding viral RNA from recognition (**Fig 4C**). If SEOV were capable of antagonizing innate immune signaling pathways, we would expect that established SEOV infection would dampen ISG expression driven by type I IFN, poly(I:C), SeV, or HTNV. Further, we expected that if SEOV were capable of inhibiting either RIG-I or MDA5 signaling, infection of cells lacking each individual RLR would lead to an inhibition of ISG expression following HTNV infection (**Fig 5E**). Importantly, we observed no such inhibition in any of these experiments, providing no evidence to support the hypothesis that SEOV actively antagonizes the RLR or type I IFN signaling cascades in reservoir EC.

A low MOI spreading infection represents a physiologically relevant scenario to study host-pathogen dynamics, with low numbers of infectious particles thought to survive the bottlenecks of host transmission and invasion to establish the initial infection [75, 79]. Despite this, it is common for *in vitro* investigations to be performed at very high, non-physiological MOI. Therefore, we investigated whether initiating infection of primary RLMVEC with high SEOV MOI could drive innate immune activation. Indeed, we observed that increasing the initial MOI led to a clear induction of ISGs, with higher MOIs driving earlier gene expression (**Fig 7A**). Notably, even at the highest MOI, ISG expression still does not occur until day 3 post-infection, suggesting that viral propagation and spread is required for immune signaling. This observation is supported by a recent report by Noack et al., reporting that SEOV infection at an MOI of 1 induced an antiviral transcriptional response in both human and rat ECs [76]. Further, we observed that ISG induction in response to high starting MOI was dependent on RIG-I (**Fig 7B**). It is well-established that high MOI infections with negative-sense RNA viruses drives the generation of defective interfering (DI) particles (also referred to as non-standard/defective viral genomes) [69,71,72,80,81]. Recently, a greater appreciation for the role of defective particles and incomplete genomes in triggering innate immune sensors has been highlighted in the literature [82]. Thus, the induction of innate immune activation in primary RLMVEC following high MOI infection is possibly due to the accumulation of DI particles. Another possibility is that high MOI infections allow for individual cells to be infected with several viral particles at once, limiting access to critical host factors that facilitate efficient replication and evasion of host PRR, leaving viral PAMPs exposed in the cytosol. Remodeling of intracellular membranes by Tula virus has been proposed as a mechanism of hantavirus sequestration of replication complexes away from surveilling PRR, such as RIG-I and MDA5 [83]. SEOV may also be capable of limiting the accumulation of unprocessed RNA or double-stranded RNA intermediates during replication. Trimming of the 5’-triphosphate to a monophosphate on the viral genome is known to be a RIG-I-specific evasion strategy employed by hantaviruses, and may require specific host interactions [84–86]. Further research will be necessary to define the mechanism driving immune activation only at high MOI, but our investigations demonstrate the important nuance in interpreting viral data performed only at very high or very low MOI.

We have yet to determine the mechanism(s) by which SEOV limits innate immune activation in low starting MOI conditions, despite strong protein expression and high susceptibility to infection.

A deeper understanding of the global SEOV interactome within reservoir and non-reservoir hosts to identify critical protein-protein associations will inform future investigations into these potential host-specific evasion mechanisms. Investigations, like this one, aiming to define the virus-host interactions that allow highly pathogenic zoonotic RNA viruses to be maintained within wild reservoirs are critical to understand the underlying mechanisms of reservoir persistence, as well as provide novel avenues for antiviral therapeutic development.

## Materials and methods

### Viruses and *in vitro* infections

Seoul virus strain SR11 and Hantaan virus strain 76–118 were propagated on Vero E6 cells (ATCC, CRL-1586) for 12 days. Infectious virus was isolated by harvesting supernatant and centrifuging at 1000rcf for 10 minutes to remove cellular debris. Rat-passaged SEOV was generated using SR11 stock and propagated over three passages (MOI 0.01) in RLMVECs for ten days each. SEOV Baltimore strain, kindly provided by Dr. Steven Bradfute (UNM), was generated in Vero E6 cells and harvested 12 days post-infection. Sendai virus/52 was purchased from ATCC (Sendai/52, VR-105). For virus infections, cells were seeded in cell culture vessels 18–24 hours prior to infection at a target density of 70%. Virus stock was diluted to the target, cell-specific MOI/HAU using serum-free Dulbecco’s modified Eagle’s medium (DMEM, VWR 45000–304) supplemented with 1x pen/strep, 1% nonessential amino acids, 2.5% HEPES, and cells were infected for one hour at 37°C. Cells were washed twice with sterile PBS solution (FisherScientific, SH30264FS), and appropriate culture medium was added for the duration of the experiment. For infected cell RNA experiments, Vero E6 cells were infected with SEOV for five days at MOI 0.5 prior to harvest in TRIzol reagent and subsequent RNA extraction. Percent SEOV N positive and infectious unit quantification experiment was performed using SEOV stock virus derived from Vero E6 cells, 1:2 dilution series in serum-free media was performed and cells were infected in a 96-well plate (Agilent, 204624–100) for 1 hour at 37°C followed by addition of methylcellulose (Sigma M0512-500G) overlay with supplemented at 2x concentration DMEM (Gibco, 12100–046) supplemented with 2% FBS, 1x PenStrep, 1% HEPES. UV inactivated virus was treated with UV radiation (FisherBrand UV crosslinker, 13-245-221) until specific absorption of 103.8 mJ/cm^2^.

### Dryad Doi

doi:10.5061/dryad.gf1vhhmzd [92]

### Cell culture

Vero E6 cells (ATCC, CRL-1586) and HEK293T (ATCC, CRL-3216) cells were cultured in Dulbecco’s modified Eagle’s medium (DMEM) supplemented with 10% heat-inactivated FBS, 1% pen/strep, 1% nonessential amino acids, 2.5% HEPES. Primary rat microvascular endothelial cells (RLMVEC, VEC Technologies) were cultured in MCDB-131 base medium (Corning) supplemented with EGMTM-2 Endothelial SingleQuots Bullet Kit (Lonza, CC-4176) and 10% heat-inactivated FBS. Human umbilical endothelial cells (HUVEC-C; ATCC, CRL-1730) were cultured in Lonza EGM-Plus (Lonza, CC-4542) supplemented with bullet kit (Lonza, CC-5036) and 10% heat-inactivated FBS. All endothelial cells were cultured in tissue culture-treated plastics coated with rat tail collagen (VWR, 47747–218). HUVEC RLR CRISPR knockout lines were generated and validated previously [38]. Recombinant rat IFNβ was purchased from R&D Systems (13400–1) and used at 10-150U/milliliter. RLMVEC knockout cells were generated using LentiCrispr V2 plasmid system [87] with gRNAs targeting RIG-I, MDA5, or Cas9 Scramble as control (Table 1). Briefly, HEK293T cells were transfected using ProFection Mammalian Transfection System (Promega, PAE1200) with targeted LentiCrispr V2 plasmid (Addgene Plasmid #98290), pSPAX (Addgene #12260), and p-VSVG (Addgene #138479). One day post-transfection, fresh DMEM was added to HEK293T cells. Virus was harvested two days post-transfection from HEK293T supernatant, filtered through a 0.45μm filter, and added to RLMVECs for 24 hours. Single knockout cells were placed under puromycin (VWR, AAJ67236-8EQ) selection at 1μg/milliliter for one week. RLR knockout cells were generated using the opposite RLR gRNA (e.g., RLMVEC RIG-I^-/-^ with puromycin resistance, then treated with MDA5^-/-^ gRNA with blastocydin resistance) in a LectiCrispr V2 plasmid encoding blastocydin selection and placed under selection (10μg/milliliter) for 1 week. Knockout efficacy was verified via treatment with rat recombinant IFNβ and immunoblot (**S5 Fig**).

**Table 1:** gDNA sequences for RLMVEC CRISPR knockout lines.

Target	Domain	gDNA Sequence
Non-target	N/A	GACGGAGGCTAAGCGTCGCAA
RIG-I	Exon 1	CAGCTATATGAGTTCCTGGC
MDA5	Exon 2	TCCTGGATGTTCTTCGCCAA

### Focus forming unit assay

Infectious virus was quantified using immunostaining as previously described [60]. Briefly, Vero E6 cells were infected with 100μL of culture supernatants and incubated for two hours at 37°C. After incubation, a 2% carboxymethylcellulose overlay containing supplemented DMEM (2% pen/strep, 2% nonessential amino acids, 2% HEPES, 4% heat-inactivated FBS). Cells were incubated for seven days at 37°C. After incubation, cells were fixed with 95% EtOH:5% Acetic Acid for 10 minutes at -20°C. Cells were then probed for SEOV N (α SEOV nucleocapsid, custom generated with Genscript) or HTNV N (anti-HTNV nucleocapsid 76–118, BEI resources NR-12152) at room temperature for 2 hours or 4°C overnight. HRP conjugated secondary antibodies (donkey α rabbit for α HTNV, Jackson Immunoresearch 711-035-152; or goat α mouse for α SEOV, Jackson Immunoresearch 115-035-003) were incubated for 2 hours at room temperature. Foci were then stained using Vector VIP Substrate Kit (Vector Laboratories, SK-4600) and counted under a light microscope to calculate titer.

### Immunofluorescence and microscopy analysis

To calculate SEOV N positivity and cell-specific titers, Vero E6, HUVEC, and RLMVEC were cultured in 96-well plates and infected with serial dilutions of SEOV as described in infection methods above. 24 hours post-infection, cells were fixed with 95% EtOH:5% Acetic Acid solution for ten minutes at -20°C then blocked for one hour in 3% FCS in PBS. SEOV N positive RLMVECs at 0.05, 0.1, and 0.25 MOI timecourse were cultured in 6-well plates and fixed and blocked in the same manner as the 96-well plate assay. Cells were probed overnight at 4°C for SEOV N protein (custom, Genscript) at dilution 1:400 in 1xPBS and with secondary antibody AlexaFluor 555 goat α mouse (Thermo Fisher Scientific, A-31570) at dilution 1:400 for two hours at room temperature. Nuclear stain DAPI was used at 1:10,000 for ten minutes at room temperature (SeraCare, 5930–0006). High-content imaging was acquired on CellInsight CX7 High-content Analysis platform (Thermo Fisher Scienitific, CX7A1110) at 10x objective magnification using iDev software. SEOV N positive cells were quantified using the Spot Detector Applet in the iDev software. Regions of interest (ROI) were defined based on DAPI nuclear stain, and SEOV N positive cells were defined as having at least one spot within the ROI as thresholded relative to each cell type. Data were collected on a total of 25 fields per well for all cell types for 96-well plate, or 250 fields per well for 6-well plates. Representative images were acquired on the EVOS FL Auto imaging system (Thermo Fisher Scientific, AMF7000) at 10x magnification for 96-well plate, or 20x magnification for MOI series 6-well plate. RLMVECs assayed for Mx1/2/3 expression during SEOV infection were fixed using 4% paraformaldehyde (VWR, 97064–606) solution in sterile PBS for 30 minutes at room temperature, then permeabilized with 0.01% Triton X-100 (VWR, 97062–208) and blocked for one hour in 3% FCS in PBS. Cells were treated with DAPI nuclear stain 1:1000, SEOV N nucleocapsid 1:400, or Mx 1/2/3 1:400 in PBS (Santa Cruz Biotechnology, sc-166412 AF488). Representative images were acquired via the EVOS FL Auto imaging system at 20x magnification (Thermo Fisher Scientific). The entire dataset that contributed to the final analysis shown can be found at https://datadryad.org/stash/dataset/doi:10.5061/dryad.gf1vhhmzd [92].

### Cell-specific titer calculations

Cell-specific titers were calculated by determining the number of SEOV N-expressing cells for each cell type using a serial dilution of SEOV stock. This method is adapted from a recent publication by Menke et al. [61]. Briefly, total number of N expressing cells was quantified as described above. For each dilution:

((%Npositivecells÷100)xtotalnumberofcellsidentifiedx13.3)xdilution(ex.1:10)=infectiousunits/milliliter

Titer was calculated for all replicate wells in each dilution for which the number of N-positive cells decreased two-fold, to match the inoculum dilutions. Replicates were averaged and then the calculated titers for each dilution condition were averaged to determine the titer on each cell type. Four replicates per dilution and >3 independent experiments were performed for each cell type. The entire dataset that contributed to the final analysis shown can be found at https://datadryad.org/stash/dataset/doi:10.5061/dryad.gf1vhhmzd [92].

### RNA Methods

Cells were lysed for RNA analysis using TRIzol Reagent (ThermoFisher Scientific, 15596026). RNA used for infected cell RNA transfection was isolated using phenol:chloroform extraction following manufacturer protocol. RNA used for gene expression analysis was purified using Zymo Research Direct-zol RNA Miniprep Plus kit (VWR 76211–340) according to manufacturer’s instructions. RNA concentrations were quantified using a NanoDrop UV-Vis Spectrophotometer (ND-1000). cDNA was synthesized using Applied Biosystems High-Capacity cDNA Reverse Transcription Kit (FisherScientific, 43-688-14) by adding 400ng total RNA to the reaction mixture containing random primers following manufacturer guidelines. Real-time PCR and quantitative-Real-time PCR was performed using the SYBR Green PCR Master Mix (ThermoFisher Scientific, 4364346) and the primers described in Table 1. In-house designed primers were purchased from Integrated DNA Technologies and TaqMan primer assays were purchased from ThermoFisher. Host antiviral ISG expression and SeV gene expression were assessed with primers in Table 2 and normalized to rat Chmp2a mRNA, and fold change calculated as ddCt over mock infection. Viral RNA copies for SEOV were calculated using a DNA plasmid standard encoding the N gene and the noted primers during qRT-PCR. Results were visualized and statistically analyzed using GraphPad Prism software (Prism 10). The entire dataset that contributed to the final analysis shown can be found at https://datadryad.org/stash/dataset/doi:10.5061/dryad.gf1vhhmzd [92].Synthetic polyinosinic:polycytidylic acid (poly(I:C)) was purchased from Sigma-Aldrich (MilliporeSigma, P0913-50MG). The hepatitis C virus (HCV) xRNA was *in vitro* transcribed from synthetic DNA oligonucleotide templates (Integrated DNA Technologies) using the T7 MEGAshortscript kit (Ambion) as previously described [88–90]. Following manufacturer protocol, transfections were completed using JetPRIME transfection reagent (VWR, 89129–924).

**Table 2:** Primer sequences or catalog numbers for real-time PCR analyses.

Species	Target	Sequence
Rat	*Chmp2a*	AGACGCCAGAGGAACTACTTC
		ACCAGGTCTTTTGCCATGATTC
Rat	*Cxcl10*	TGAAAGCGGTGAGCCAAAGA
		CTAGCCGCACACTGGGTAAA
Rat	*Ccl2*	AGCCAACTCTCACTGAAGC
		GTGAATGAGTAGCAGCAGGT
Rat	*Rig-i*	GGAAGTGATCTTACCTGCTCTGG
		TTGCCTCTGTCTACCGTCTCT
Rat	*Ifn*β	CACATTGCGTTCCTGCTGTG
		AGTGCTTTGTCGGAACTGGA
Rat	*Chmp2a*	TaqMan Gene Expression Assay (FAM) Rn01520393_m1
Rat	*Mx1*	TaqMan Gene Expression Assay (FAM) Rn00597360_m1
Rat	*Oas1*	TaqMan Gene Expression Assay (FAM) Rn04219673_m1
Rat	*Ifit3*	TaqMan Gene Expression Assay (FAM) Rn01479928_m1
SEOV	Nucleocapsid	GTCGGAGGGATGGCTGAAT
		CCACAGTTTTTGAAGCCATGATT
HTNV	Nucleocapsid	AAGCATGAAGGCAGAAGAGAT
		TAGTCCCTGTTTGTTGCAGG
SeV	Genome position 332–307	TCTCTGAGAGTGCTGCTTATCTGTGT
	Genome position 210–233	CAGAGGAGCACAGTCTCAGTGTTC

### Protein methods

Cell lysates to be analyzed for protein expression by immunoblot analysis were harvested in protein lysis buffer and prepared as previously described [91]. Briefly, protein was harvested in RIPA buffer and clarified through 25,000rcf centrifugation for 15 minutes at 4°C. Protein was quantified via Pierce BCA Protein Assay Kit (ThermoFisher Scientific, 23225). 15μg-30ug of protein was loaded per sample into a 10% polyacrylamide gel and, after denaturing electrophoresis, transferred to a 0.45μm nitrocellulose membrane (VWR, 10120–006). Membranes were blocked at room temperature in 10% FCS in PBS-T. Primary antibodies (Table 3) were incubated at 4° overnight. HRP-conjugated secondary antibodies against primary antibody species were incubated for 1 hour at room temperature. Blots were imaged on BioRad Chemidoc MP Imaging System using chemiluminescence Pierce Substrate for Western Blotting (VWR, PI80196). The entire dataset that contributed to the final analysis shown can be found at https://datadryad.org/stash/dataset/doi:10.5061/dryad.gf1vhhmzd [92].

**Table 3:** Primary antibodies used for protein detection in immunoblot and immunofluorescence assays.

Target Species	Protein	Manufacturer
Rat	Mx 1/2/3	SantaCruz Biotech (sc-166412)
Rat	Mx 1/2/3 AF488	SantaCruz Biotech (sc-166412 AF488)
Rat	MDA5	GeneTex (GTX103138)
Rat	RIG-I	Cell Signaling Technologies (3743T)
Human, Rat	βActin	SantaCruz Biotech (sc-47778)
Human	Mx	Cell Signaling (37849S)
Human	IFIT1	Abcam (ab236256)
SEOV	SEOV Nucleocapsid	Custom, Genscript

### Densitometry Quantification of Immunoblots Using ImageJ

Densitometry analysis of immunoblots was performed using ImageJ (NIH) to quantify protein band intensities. Immunoblot images were first converted to 8-bit grayscale. The rectangular selection tool was used to draw a box around each band, ensuring consistent box sizes across all bands. The area under the curve (AUC) for each band was measured using the “Analyze → Gels → Plot Lanes” function, which generates intensity profiles of the bands. The peak areas corresponding to each band were selected using the wand tool, and background intensity was subtracted from each lane. The relative band intensities were normalized to the loading control (β-actin) to correct for variations in protein loading. Data from presented experiments were reported as relative protein expression levels compared to β-Actin. The entire dataset that contributed to the final analysis shown can be found at https://datadryad.org/stash/dataset/doi:10.5061/dryad.gf1vhhmzd [92].

### Secreted cytokine analysis

Tissue culture supernatant from HUVEC (Cas9 and RLR^-/-^) was collected four days post SEOV infection (MOI 0.01) and clarified for cellular debris by centrifugation (1000 rcf for 10 minutes). Clarified supernatants were UV-inactivated (specific absorption of 103.8 mJ/cm^2^) and shipped to Eve Technologies for interrogation on the Human Cytokine/Chemokine Panel A 48-Plex Discovery Assay Array (HD48A). One experiment was performed with three biological replicates and two technical assay replicates.

### Statistical analyses

Statistical significance was assess using GraphPad Prism software version 10. Where noted in figure legends, ANOVA and student’s T-test analyses were applied, with * representing adjusted p<0.05. All comparisons reaching statistical significance are displayed on graphs.

## Supporting information

S1 Fig Expanded Cytokine Panel for SEOV Infected HUVEC Cas9 and RLR^-/-^. An expanded panel of cytokine detection results from Fig 2B.
(XLSX)

S2 Fig Densitometry of Fig 2 immunoblots. Densitometry quantification of immunoblot in Fig 2A.
(TIF)

S3 Fig SEOV Baltimore Strain Does Not Induce ISG Response in Primary Rat EC. A) RLMVEC were infected with either SEOV Baltimore strain (SEOV B) (MOI 0.05), UV-inactivated SEOV Baltimore strain (UV SEOV B) (MOI 0.05) or HTNV (MOI 0.05). Lysates were harvested at the indicated times post-infection and subjected to immunoblot analysis. B) Densitometry of immunoblot in panel A.
(TIF)

S4 Fig Densitometry of Fig 3 Immunoblots. A) Densitometry quantification of immunoblot in Fig 3A. B) Densitometry quantification of immunoblot in Fig 3C. C) Densitometry quantification of immunoblot in Fig 3D.
(TIF)

S5 Fig Validation of RLMVEC MDA5^-/-^, RIG-I^-/-^, or RLR^-/-^. Wild type, Cas9 scramble, MDA5^-/-^, RIG-I^-/-^, and RLR^-/-^ RLMVECs were either mock-treated or treated with 150 U/mL of recombinant rat IFNβ for 24 hours. Lysates were collected and subjected to SDS-PAGE followed by immunoblot analysis to verify the effective knockout of the target genes.
(TIF)

S6 Fig Densitometry of Fig 5 Immunoblots. A) Densitometry quantification of immunoblot in Fig 4B. B) Densitometry quantification of immunoblot in Fig 4E.
(TIF)

S7 Fig High MOI infections drive RLR-dependent ISG expression in RLMVEC. A) RLMVEC were infected with SEOV at the noted MOI 0.05–0.75 and cell lysates collected every 24 hours, as indicated. Cell lysates were subjected to immunoblot analysis. Mock-infected RLMVEC (-) treated with 100U/mL IFNβ 24 hours prior to harvest serves as a positive control. B) Cas9 scramble, RIG-I^-/-^, MDA5^-/-^, or RLR^-/-^ RLMVEC were mock-infected (-) or infected with SEOV at either MOI 0.05 or 0.5 and harvested at the indicated times post-infection. Lysates were subjected to immunoblot analysis. Densitometry quantification of immunoblots in S8 Fig. All data shown represent ≥3 independent experiments.
(TIF)

S8 Fig Densitometry of Fig 7 Immunoblots. A) Densitometry quantification of immunoblot in Fig 7A. B) Densitometry quantification of immunoblot in Fig 7B.
(TIF)
