# Supplementary figures and images for: Seoul orthohantavirus evades innate immune activation by reservoir endothelial cells
Source: PLoS Pathog. 2024 Nov 25;20(11):e1012728. doi: 10.1371/journal.ppat.1012728 (PMC11627401; doi:10.1371/journal.ppat.1012728)

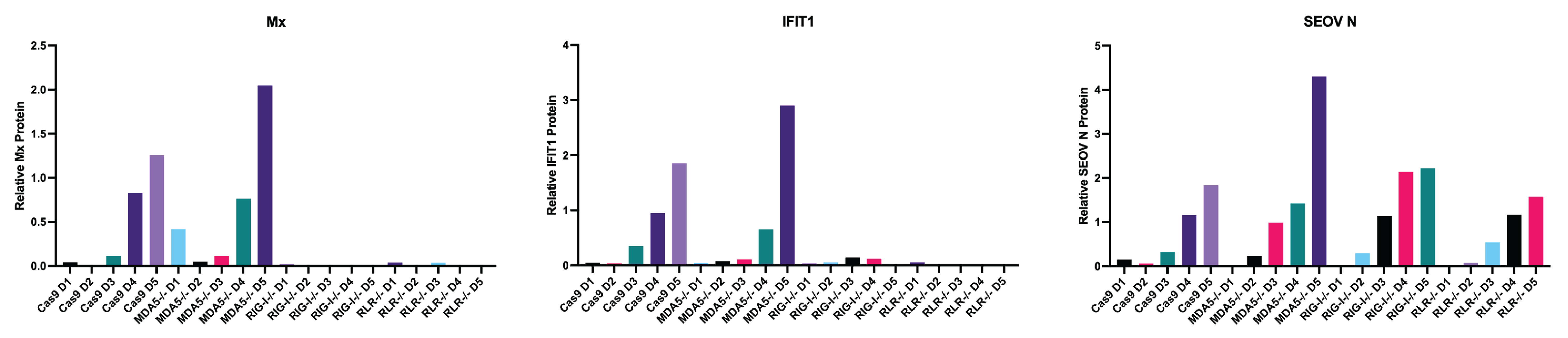

Supplement: S2 Fig — Densitometry quantification of immunoblot in Fig 2A. (TIF) [file ppat.1012728.s002.tif]

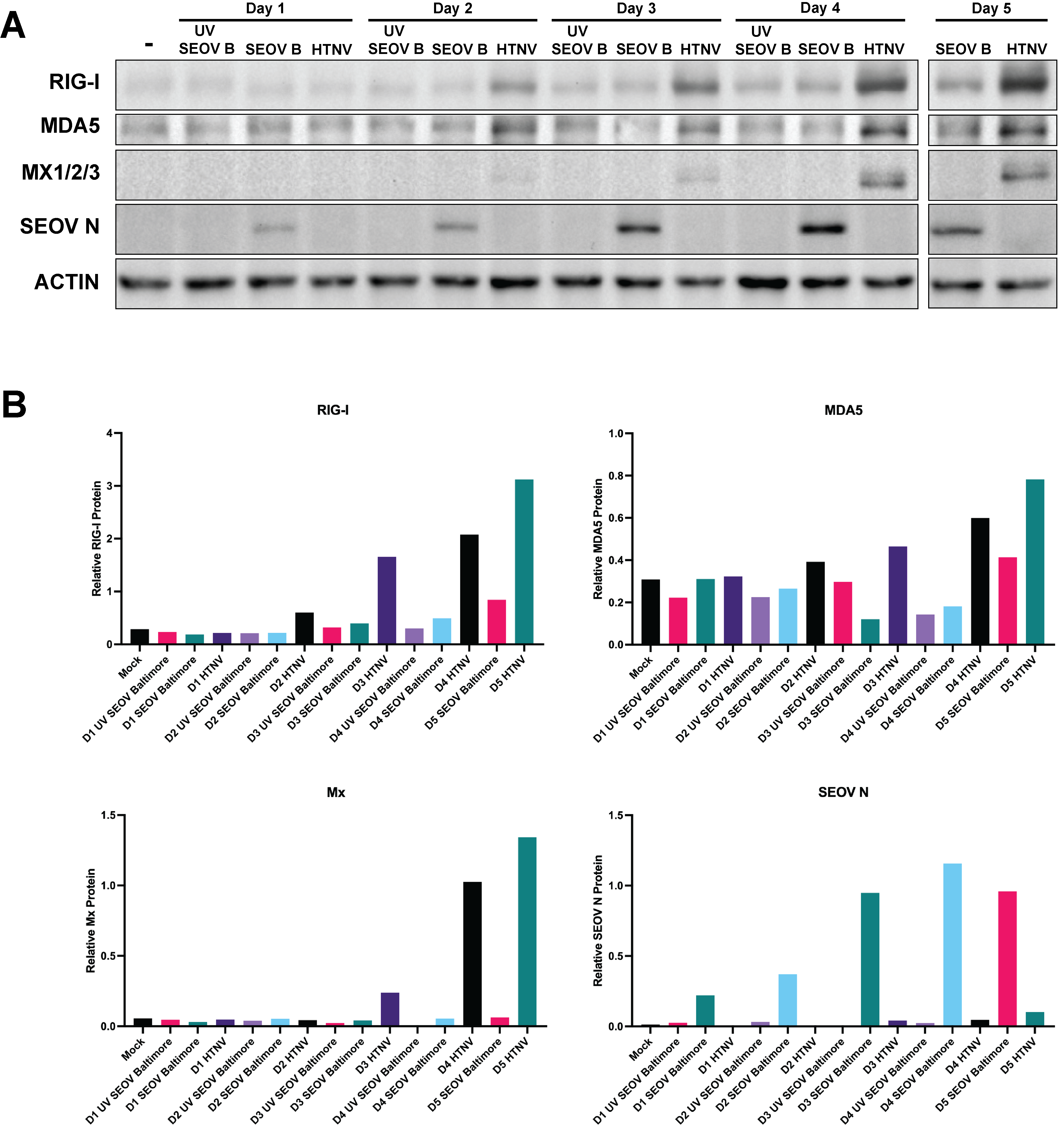

Supplement: S3 Fig — A) RLMVEC were infected with either SEOV Baltimore strain (SEOV B) (MOI 0.05), UV-inactivated SEOV Baltimore strain (UV SEOV B) (MOI 0.05) or HTNV (MOI 0.05). Lysates were harvested at the indicated times post-infection and subjected to immunoblot analysis. B) Densitometry of immunoblot in panel A. (TIF) [file ppat.1012728.s003.tif]

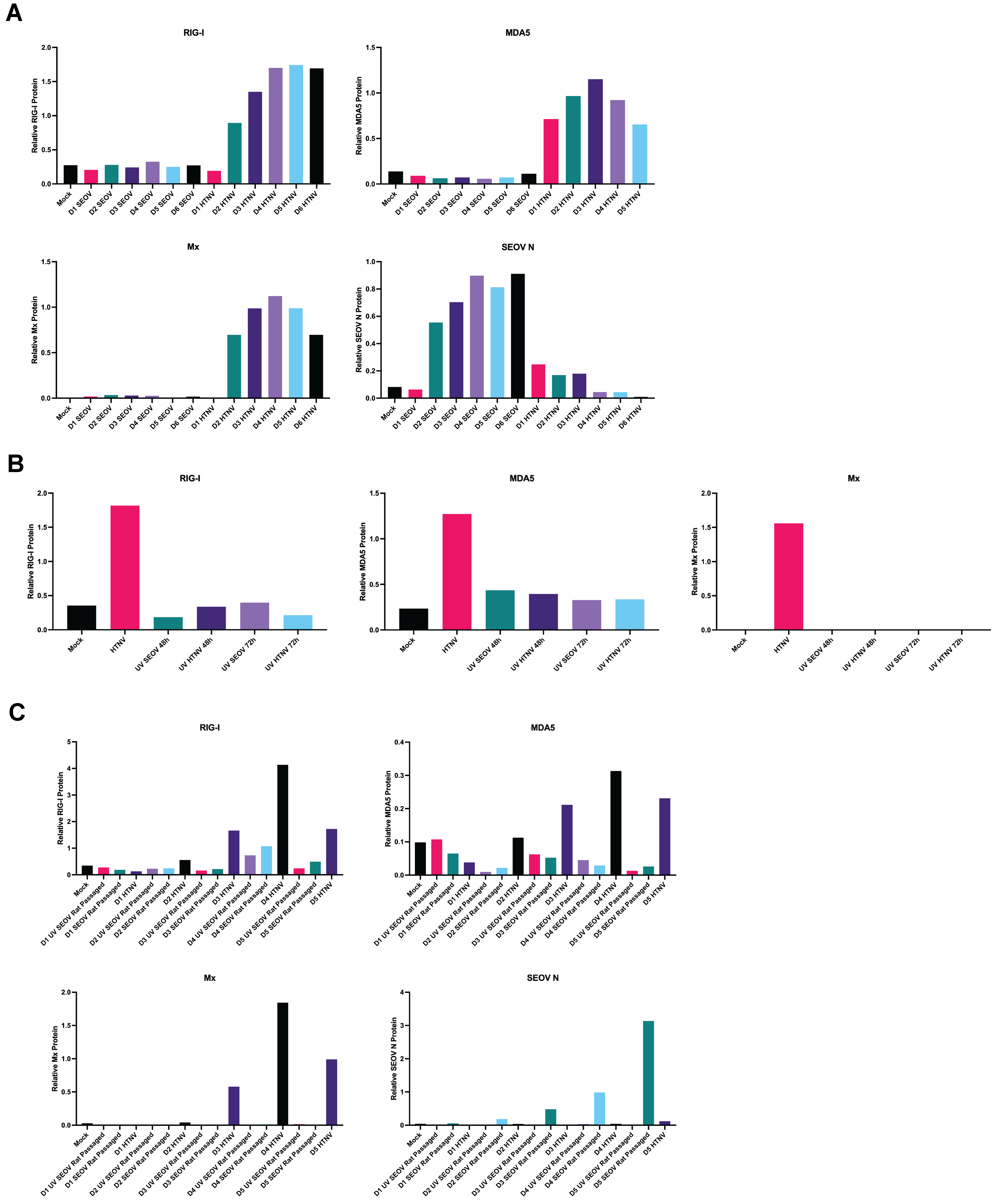

Supplement: S4 Fig — A) Densitometry quantification of immunoblot in Fig 3A. B) Densitometry quantification of immunoblot in Fig 3C. C) Densitometry quantification of immunoblot in Fig 3D. (TIF) [file ppat.1012728.s004.tif]

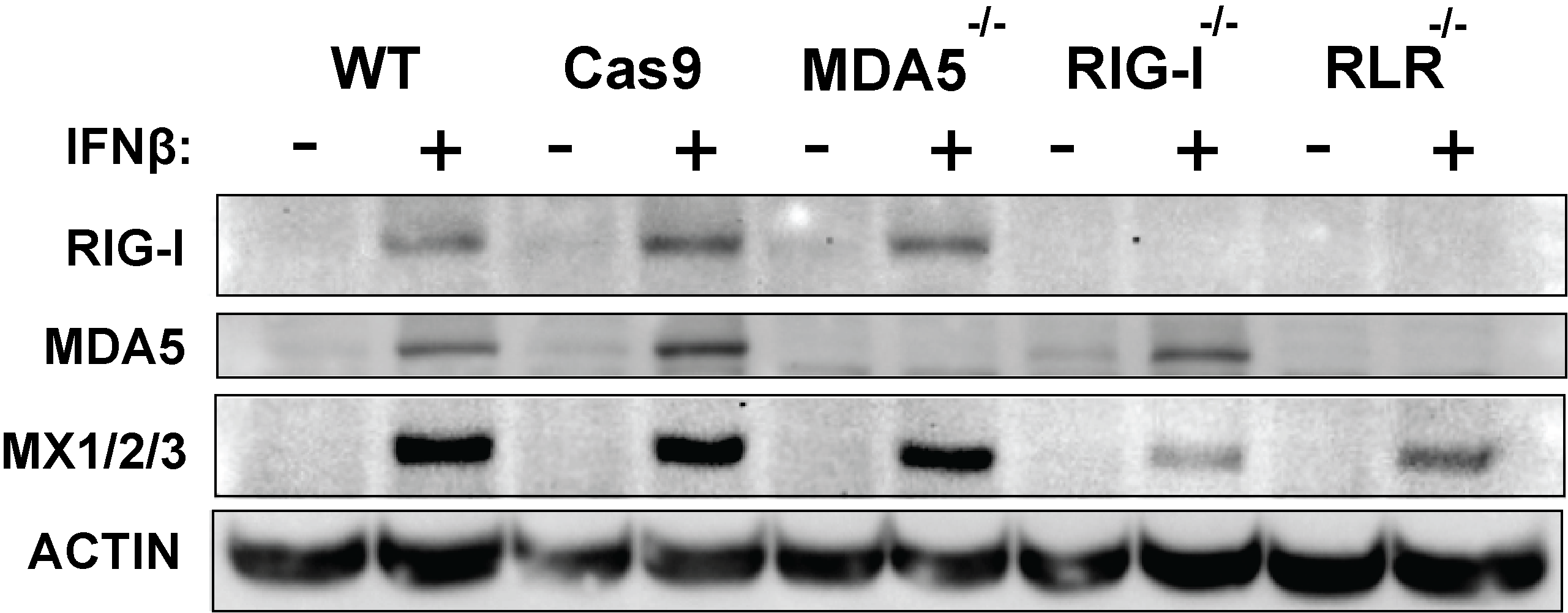

Supplement: S5 Fig — Wild type, Cas9 scramble, MDA5-/-, RIG-I-/-, and RLR-/- RLMVECs were either mock-treated or treated with 150 U/mL of recombinant rat IFNβ for 24 hours. Lysates were collected and subjected to SDS-PAGE followed by immunoblot analysis to verify the effective knockout of the target genes. (TIF) [file ppat.1012728.s005.tif]

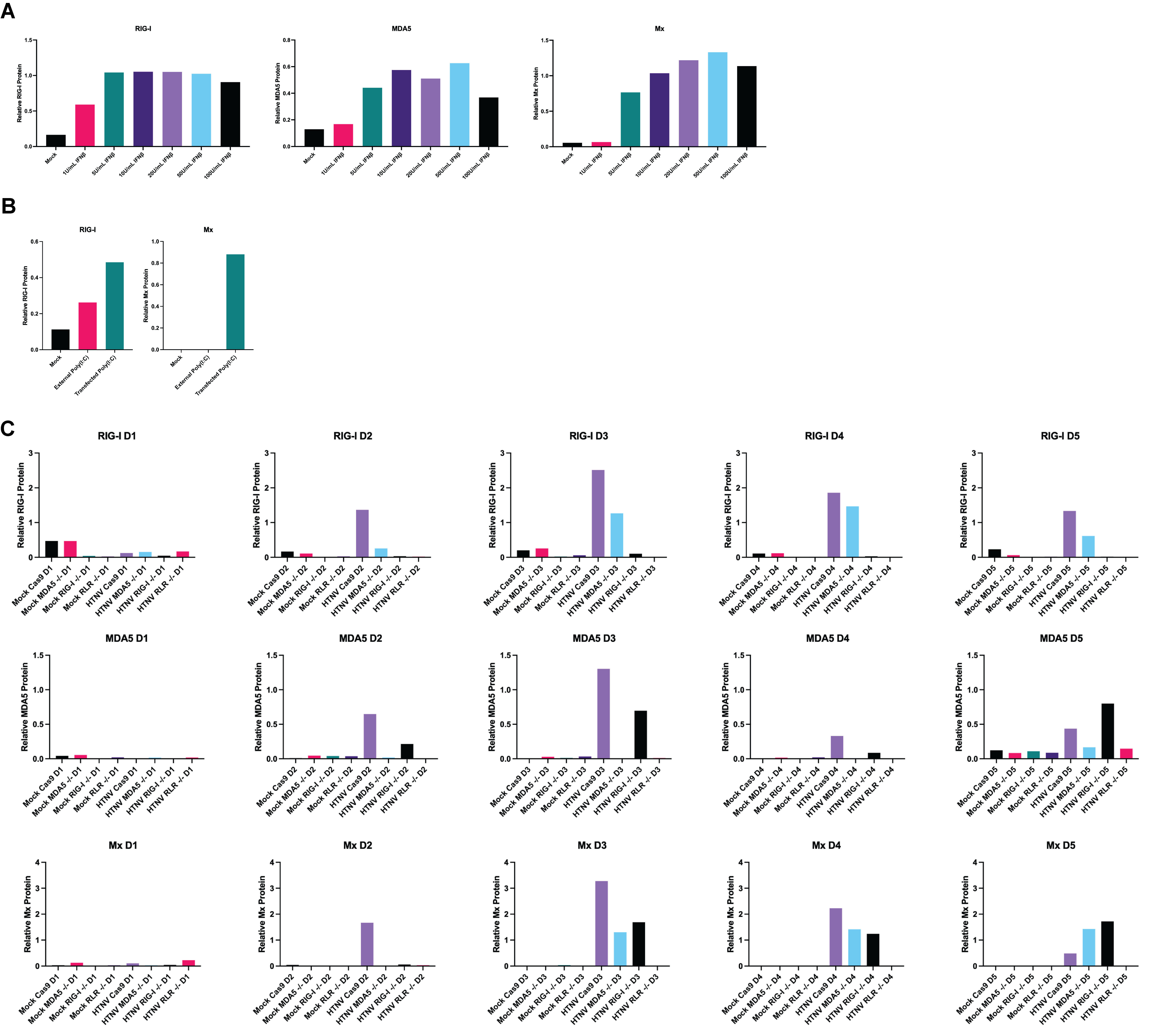

Supplement: S6 Fig — A) Densitometry quantification of immunoblot in Fig 4B. B) Densitometry quantification of immunoblot in Fig 4E. (TIF) [file ppat.1012728.s006.tif]

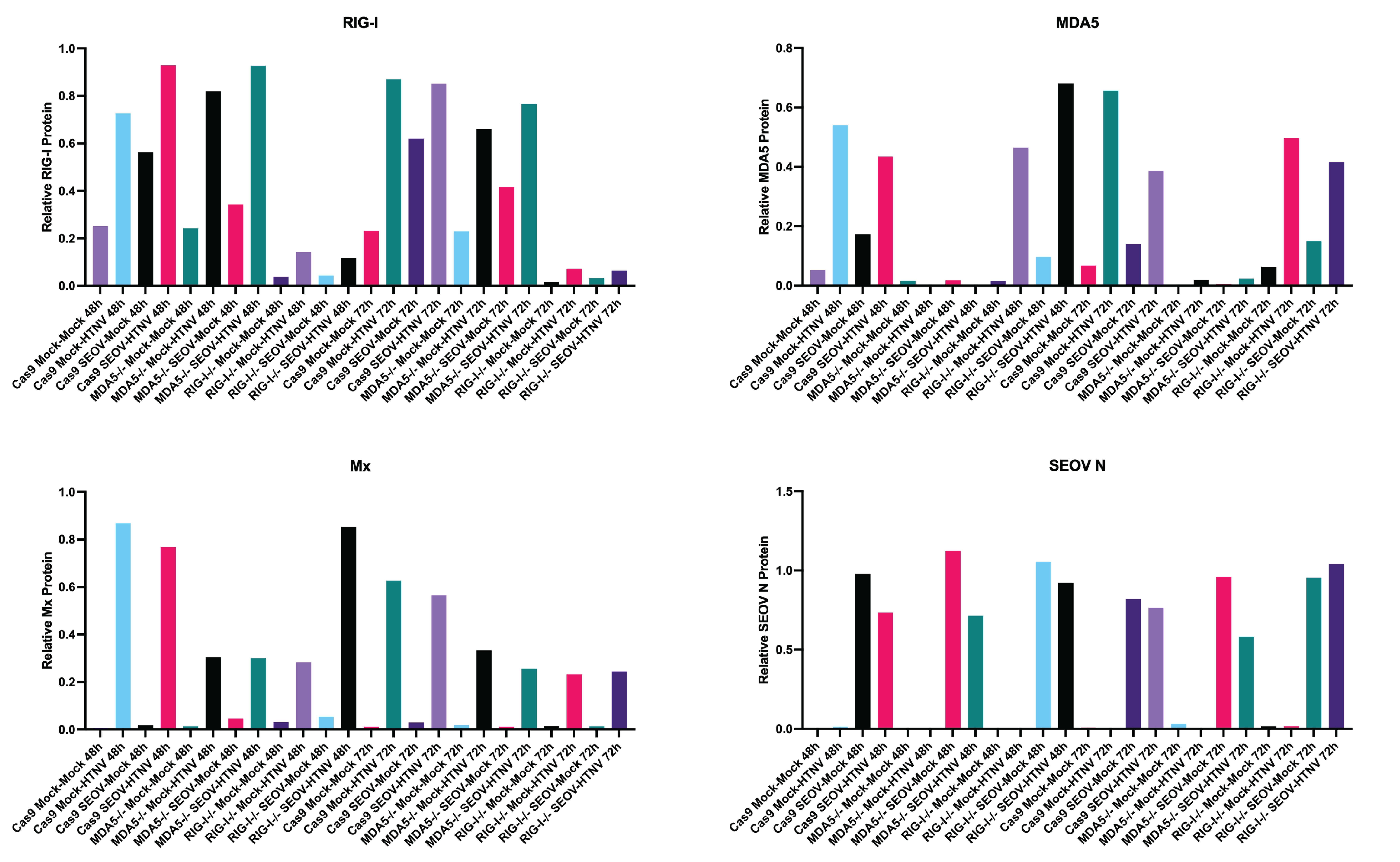

Supplement: S7 Fig — A) RLMVEC were infected with SEOV at the noted MOI 0.05–0.75 and cell lysates collected every 24 hours, as indicated. Cell lysates were subjected to immunoblot analysis. Mock-infected RLMVEC (-) treated with 100U/mL IFNβ 24 hours prior to harvest serves as a positive control. B) Cas9 scramble, RIG-I-/-, MDA5-/-, or RLR-/- RLMVEC were mock-infected (-) or infected with SEOV at either MOI 0.05 or 0.5 and harvested at the indicated times post-infection. Lysates were subjected to immunoblot analysis. Densitometry quantification of immunoblots in S8 Fig. All data shown represent ≥3 independent experiments. (TIF) [file ppat.1012728.s007.tif]
